# eRegistries: indicators for the WHO Essential Interventions for reproductive, maternal, newborn and child health

**DOI:** 10.1186/s12884-016-1049-y

**Published:** 2016-09-30

**Authors:** Vicki Flenady, Aleena M. Wojcieszek, Ingvild Fjeldheim, Ingrid K. Friberg, Victoria Nankabirwa, Jagrati V. Jani, Sonja Myhre, Philippa Middleton, Caroline Crowther, David Ellwood, David Tudehope, Robert Pattinson, Jacqueline Ho, Jiji Matthews, Aurora Bermudez Ortega, Mahima Venkateswaran, Doris Chou, Lale Say, Garret Mehl, J. Frederik Frøen

**Affiliations:** 1 Mater Research Institute, The University of Queensland (MRI-UQ), Brisbane, Australia; 2 International Stillbirth Alliance, Bristol, UK; 3 Department of International Public Health, Norwegian Institute of Public Health, Oslo, Norway; 4 Department of Epidemiology and Biostatics, School of Public Health, College of Health Sciences, Makerere University, Kampala, Uganda; 5 Centre for Intervention Science in Maternal and Child Health (CISMAC), Centre for International health, University of Bergen, Bergen, Norway; 6 South Australian Health and Medical Research Institute (SAHMRI), Adelaide, Australia; 7 Liggins Institute, University of Auckland, Auckland, New Zealand; 8 Griffith University & Gold Coast University Hospital, Gold Coast, Australia; 9 Medical Research Council, University of Pretoria, Pretoria, South Africa; 10 Penang Medical College and Penang Hospital, Penang, Malaysia; 11 Christian Medical College, Vellore, Tamil Nadu India; 12 The Australian Nurse Family Partnership Program National Program Centre, Abt Australia, Brisbane, Australia; 13 Department of Reproductive Health and Research, World Health Organization, Geneva, Switzerland

**Keywords:** Performance indicators, Process indicators, Outcome indicators, Intervention coverage, ehealth, Registries, Maternal health, Perinatal health, Newborn health

## Abstract

**Background:**

Electronic health registries – *eRegistries* - can systematically collect relevant information at the point of care for reproductive, maternal, newborn and child health (RMNCH). However, a suite of process and outcome indicators is needed for RMNCH to monitor care and to ensure comparability between settings. Here we report on the assessment of current global indicators and the development of a suite of indicators for the WHO *Essential Interventions* for use at various levels of health care systems nationally and globally.

**Methods:**

Currently available indicators from both household and facility surveys were collated through publicly available global databases and respective survey instruments. We then developed a suite of potential indicators and associated data points for the 45 WHO *Essential Interventions* spanning preconception to newborn care. Four types of performance indicators were identified (where applicable): process (i.e. coverage) and outcome (i.e. impact) indicators for both screening and treatment/prevention. Indicators were evaluated by an international expert panel against the *eRegistries* indicator evaluation criteria and further refined based on feedback by the *eRegistries* technical team.

**Results:**

Of the 45 WHO Essential Interventions, only 16 were addressed in any of the household survey data available. A set of 216 potential indicators was developed. These indicators were generally evaluated favourably by the panel, but difficulties in data ascertainment, including for outcome measures of cause-specific morbidity and mortality, were frequently reported as barriers to the feasibility of indicators. Indicators were refined based on feedback, culminating in the final list of 193 total unique indicators: 93 for preconception and antenatal care; 53 for childbirth and postpartum care; and 47 for newborn and small and ill baby care.

**Conclusions:**

Large gaps exist in the availability of information currently collected to support the implementation of the WHO *Essential Interventions*. The development of this suite of indicators can be used to support the implementation of *eRegistries* and other data platforms, to ensure that data are utilised to support evidence-based practice, facilitate measurement and accountability, and improve maternal and child health outcomes.

**Electronic supplementary material:**

The online version of this article (doi:10.1186/s12884-016-1049-y) contains supplementary material, which is available to authorized users.

## Background

Lack of quality data on the health status of individuals is a major contributor to poor health outcomes at a population level [1]. Progress towards the United Nations recently proposed Sustainable Development Goals (SDGs) [2] can only be monitored with efficient and effective health information systems. Despite this obvious need, most low- and middle-income countries (LMIC) have insufficient systems in place for the collection, analysis and reporting of health data, severely hampering both health system and policy-level decision-making [3].

Improvement of data collection is ongoing in many countries with the advent of electronic methods of data collection, including electronic medical records to replace paper-based systems. The *eRegistries* Initiative aims to support a safe and efficient transition to integrated electronic health information systems in LMIC [4, 5] (Frost M, Hodne Titlestad O, Lewis J, Mehl G, Frøen JF: eRegistries: Architecture and Free Open Source Software for maternal and child health Registries, submitted). *eRegistries* should collect and manage information that is adapted both to the health system’s information needs, as well as the data collection and real-time analysis methodology. Many existing indicators for monitoring health have been designed and constructed to be reliably measured from household and facility surveys [6, 7]; for example, breastfeeding rates for children <6 months of age. While these surveys are critical for national and global monitoring needs [8], the historically weak data-collection capabilities of LMIC [9, 10] render the utility of such indicators limited in the context of the current global transition to more robust health information systems.

Further, while these indicators are designed to guide national policy level decisions and are collected through relatively infrequent population surveys, improvement in service delivery is not their primary aim. Similarly, many facility-based surveys only include indicators to assess readiness to deliver healthcare, leaving little attention paid to quality of care. There are gaps in the specific indicators needed to monitor and improve quality of care, which are needed to supplement the existing facility readiness and the population health indicators.

The Donabedian framework [11] describes three types of indicators typically needed for monitoring the provision of healthcare: structure, process and outcome. In this framework, ‘structure’ refers to the assets needed to support health care, also known as ‘inputs’ and ‘outputs’ in monitoring and evaluation frameworks [12], and include access, supplies and personnel. Process indicators refer to actions/activities performed during the delivery of health care and may be known as coverage (or, as in monitoring and evaluation frameworks, outcome) indicators. The Donabedian framework uses ‘outcomes’ indicators to refer to the endpoint of the individual health status, community or population; these are often termed impact indicators by monitoring and evaluation frameworks. ‘Bottleneck analyses’ consider six factors to assess how to change impact: three structural (access, availability, training) and three process (initial coverage, continued coverage, effective coverage) indicators [13, 14]. Effective coverage indicators attempt to include all aspects of quality within a single final indicator that summarises the proportion of patients receiving high-quality of care.

In this second paper of *eRegistries* series, we report on indicators for use in monitoring the WHO *Essential Interventions Commodities and Guidelines for RMNCH* [15], which aim to improve quality of care and health outcomes in reproductive, maternal and newborn care.

This component of the *eRegistries* Initiative had two main aims:

1. to assess the status of global indicators by describing the data that are currently available to address the WHO *Essential Interventions,* and
2. to develop a robust suite of indicators and respective data points to monitor WHO *Essential Interventions* – both screening and management components – for use in *eRegistries*.

We considered the 45 *WHO Essential Interventions* delivered up to 6 weeks post-partum, including those for: (1) preconception and periconceptual care; (2) antenatal care; (3) childbirth care; (4) postpartum care (of the mother); (5) immediate care of the newborn; (6) neonatal infection management; and (7) care for small and ill babies.

## Methods

### Assessment of the current status of global indicators

We first assessed the extent to which the 45 WHO *Essential Interventions* [15] were being addressed in either household or facility surveys, to better understand the landscape of current data needs and gaps that our indicator development project should address. Our search focussed on the most recently reported process and outcome indicators identified from globally recognised sources. First, we examined the common international databases which compile indicators from various sources. These included the Countdown to 2015 databases, UNICEF databases, WHO observatory, and UNAIDS. These sources typically reported household surveys, primarily the large scale multi-country initiatives of the Demographic and Health Surveys (DHS) and Multiple Indicator Cluster Survey (MICS). The DHS and MICS websites were then assessed for additional information which was not yet available in the large compilations already mentioned. The average value for the most recent estimate of each indicator was calculated in Microsoft Excel (Redmond, WA).

As household surveys are not designed for, nor capable of, assessing process indicators relevant to care delivered at facilities, we also assessed the availability of indicators of key interventions directly from facility-based sources. Eight survey instruments were identified which collected data through either direct observations of patients or via medical records review; we did not consider routine health information systems as each country has their own unique set of indicators that may or may not be available in the public domain. Two published reviews [16, 17] which identified available survey tools or instruments were sourced. Surveys mentioned in these two sources that did not focus on health, or were not used in multiple LMIC, were excluded. In addition, to ensure accuracy, two facility survey programs were directly contacted for supplemental information. These facility surveys have several purposes, one of which is to assess the readiness of a facility to implement quality emergency obstetric care. The survey instruments ultimately included were: Service Provision Assessment (SPA) (2012 version), Averting Maternal Death and Disability (AMDD), the Maternal and Child Health Integrated Program Quality of Care survey (MCHIP-QoC), the Postpartum Hemorrhage Prevention and Treatment questionnaire on Active Management of the third stage of Labor (POPPHI), the World Bank’s Service Delivery Indicators tool (SDI) (2012 Kenya version), WHOs Service Availability and Readiness Assessment (SARA) (version 2.1), as well as the WHO’s surveys on Perinatal Health and on Maternal and newborn health from 2007 and 2010, respectively. All documents reviewed were the most recent versions available in November 2014, unless otherwise stated. One instrument included information on management of post-partum haemorrhage and was only included in our results as a denominator for relevant interventions. Data were extracted from website materials or survey instruments. Where information was unavailable, survey support staff were interviewed to identify the number of national facility surveys performed for the specified interventions. For each intervention, three domains related to process indicators were assessed: training and knowledge, availability of supplies (inventory), and whether the intervention was actually performed. The number of survey instruments collecting any information relevant to each of the 45 *Essential Interventions* in each of these three domains was measured through careful reading of each survey module.

#### Defining indicators and data points

For each of the 45 *Essential Interventions*, we conducted a comprehensive search of existing WHO indicators, followed by indicators from other professional bodies (Table 1). Existing indicators were adapted or developed as required by the *eRegistries* technical team with reference to the practice guidelines and training manuals cited within each WHO *Essential Intervention*, and other resources where appropriate.

**Table 1:** Major sources for identifying existing indicators

WHO and UNAIDS indicators	Other indicators
• The World Health Statistics 2011 Indicator Code Book [28] • WHO indicator registry (http://www.who.int/gho/indicator_registry/en/index.html) • WHO Global Health Observatory (http://apps.who.int/gho/data/#) • Indicator registries relevant to monitoring and evaluation for specific Essential Interventions (e.g. UNAIDS Indicator Registry: http://www.indicatorregistry.org/) • The World Health Organization’s near-miss approach for maternal health [29] • Monitoring emergency obstetric care: a handbook [30] • Inter-agency Field Manual on Reproductive Health in Humanitarian Settings [31] • Countdown to 2015 - Building a Future for Women and Children report [32] • Indicators obtained from relevant reports on monitoring and evaluation of the specific intervention (e.g., the ‘WHO World Malaria Report’) found from general search of the WHO website (http://www.who.int/en/)	• The official list of MDG Indicators (mdgs.un.org) • NICE menu of indicators: (www.nice.org.uk/standards-and-indicators) • New Zealand Guidelines Group website: (www.health.govt.nz/about-ministry/ministry-health-websites/new-zealand-guidelines-group) • National Center for Health Statistics Heath indicators warehouse (www.healthindicators.gov/Indicators/) • AHRQ National guidelines clearinghouse (guideline.gov/index.aspx) • Royal College of Obstetricians and Gynaecologists Guidelines (www.rcog.org.uk/guidelines/)

Website links correct as at July 2015, we acknowledge links may be updated in future

Four types of indicators were defined for each WHO intervention:

- *process indicator/s for screening/risk identification* (the proportion of patients for whom screening tests/risk identification measures were performed);
- *outcome indicator/s for screening/risk identification* (the proportion of patients screening positive/identified as ‘at-risk’);
- *process indicator/s for treatment/management* (the proportion of patients treated); and
- *outcome indicator/s for treatment/management* (the proportion of patients with adverse outcomes in the population).

For some interventions, screening/risk assessment indicators were not applicable. We considered screening/risk indicators not applicable where the given treatment/management was recommended to all women or babies of a given clearly defined population (e.g. Antenatal care essential package for all pregnant women; Provision of thermal care for all newborns to prevent hypothermia; Early initiation and exclusive breastfeeding).

By data points we refer to the primary data being captured at the point of care, which is the source of information for numerator or denominator. Data points that could be readily collected using an electronic form addressing each of the process and outcome indicators were included. Data items specifically measuring each indicator (numerator and denominator) were developed balancing specificity and feasibility by country resource level. To maximise the feasibility of data collection, for defined conditions such as preeclampsia, simple data points referring to the diagnosis (yes/no) of the condition were accepted, rather than delineated by the individual components of the clinical and laboratory diagnosis.

#### Evaluation and refinement of indicators

Evaluation and refinement of the indicators occurred via two stages: 1) Expert panel evaluation and; 2) Response to feedback and refinement within the *eRegistries* technical team.

#### Expert panel evaluation

An international group of 47 experts in maternal and child health was assembled via the network of the International Stillbirth Alliance. Thirty-four panel members were invited to participate in consultation round 1; 35 in consultation round 2; and 44 in consultation round 3. Invited evaluators included researchers, senior clinicians and academics, obstetricians, neonatologists, maternal-fetal medicine specialists, epidemiologists, consumer advocates and others.

The *eRegistries* indicator evaluation tool assessing 10 domains was developed. The domains were derived by the *eRegistries* technical team after reviewing several existing indicator evaluation frameworks, including the New Economics Foundation AIMS criteria for indicators [18, 19], the Agency for Healthcare Research and Quality standards by which to judge quality indicator performance [20], Indicators to Monitor Maternal Health Goals [21], and the SMART criteria [22]. The evaluation tool (Additional file 1) was simplified based on pilot testing with a subsample of the expert panel, culminating in the final evaluation tool assessing the below five domains.

*eRegistries* indicator evaluation criteria:

- *Action focused:* “It is clear what needs to be done to improve outcomes associated with this indicator (e.g., immunised with tetanus toxoid to reduce neonatal tetanus)”
- *Important:* “The indicator and the data generated will make a relevant and significant contribution to determining how to effectively respond to the problem”
- *Operational:* "The indicator is quantifiable; definitions are precise and reference standards are developed and tested or it is feasible to do so"
- *Feasible:* “It is feasible to collect data required for indicator in the relevant setting”
- *Simple and valued:* “The people involved in the service can understand and value indicator”

Panel members were asked to indicate via categorical response their agreement with each of five statements addressing the above domains *(Yes/Probably/Unsure/Possibly/No/Do not wish to respond).* A comment box was provided for each indicator to enable detailed feedback. Panel members were invited to suggest other indicators or adjustments to the existing indicators. Data were analysed descriptively in Microsoft Excel by tallying the number of responses for each response category.

Indicators were evaluated in three consultation rounds: (1) Preconception/periconceptual care and antenatal care; (2) Childbirth care and postpartum care (of the mother) and; (3) Immediate newborn care, neonatal infection management, and care for small and ill babies. Additional file 2 presents a list of interventions addressed, including the number of indicators within each intervention, and where the indicators were sourced. Panel members were assigned 3 to 4 interventions in each round each and asked to evaluate all indicators within the given intervention. Interventions were assigned to evaluators randomly, unless the panel member indicated a preference based on their area of expertise.

For each intervention the panel members were given a detailed breakdown of the indicators which included definitions, numerators and denominators, data points, and references. An evidence summary for the interventions was provided based on the evidence cited in the Essential Interventions (predominantly Cochrane systematic reviews). Panel members were given an evaluation sheet along with a separate document containing background information and evaluation instructions, including further detail on the *eRegistries* indicator evaluation tool development. Evaluation materials were sent to and returned by panel members by email.

We adopted a quasi-anonymous approach for indicator evaluation; that is, while individual panel members may have known the names of other members in the group, individual responses were not identifiable to the group and panel members were not aware which interventions had been assigned for evaluation to whom.

#### Response to feedback and refinem*e*nt within the eRegistries technical team

Following descriptive analyses, indicators that consistently failed to meet (or ‘probably’ meet) the defined criteria were amended based on the evaluators’ comments, or else removed if deemed unnecessary for effective monitoring and evaluation of the given intervention. A series of meetings of the *eRegistries* technical team was held to review the updated indicators to ensure consistency in nomenclature across indicators and their data points, numerators and denominators.

#### Graphical display of potential utilisation of the eRegistries indicators

To inform the plausible utilisation of these indicators, a power graph was created reflecting different use cases. The power to detect a significant change in a given indicator was graphed in association with the indicator prevalence and a given sample size. Three scenarios of different sample sizes were assumed: 200 births annually, 10,000 births annually and 500,000 births annually, to reflect a typical rural clinic, a typical district and a typical LMIC. The graphed indicator prevalence ranged from 75 % to 0.01 %. The most likely value for each of the indicators was calculated (Additional file 3) and placed alongside the graph.

## Results

### Assessment of the current status of global indicators

Of the 45 WHO *Essential Interventions*, only 16 were addressed in any of the household survey data available. Of these 16 interventions, only 7 had more than one indicator for screening and management available. In addition to the indicators themselves, data were often available on contact between a woman and the health system during either antenatal care (one or four antenatal care visits) or during care at birth (either facility delivery or skilled birth attendance) (Table 2, population data). For example, data were available for caesarean section, ANC visits, skilled birth attendance, and facility delivery from 70, 73, 75, and 75 countries, respectively, with at least 75 % of these data from 2010–2015.

**Table 2:** Current status of use of global indicators for the 45 included WHO Essential Interventions

Intervention		Population data (Percent of the population)				Facility data (Number of survey instruments)		
		Screening process indicator	Screening outcome indicator	COVERAGE/Management process indicator	Management outcome indicator	Availability of supplies	Staff ever trained	Ever performed at this facility
Preconception/ Periconception and Pregnancy	Folic acid supplementation or fortification	NA	NA	–	–	5 of 7^i^	4 of 7	3 of 7
	Family planning^a^	–	22 %	31 %	4 %	5 of 7	4 of 7	3 of 7
	Prevention and management of STIs and HIV	–	–	–	–	1 of 7	2 of 7	2 of 7
	Safe abortion services	–	–	26 %	–	4 of 7	2 of 7	4 of 7
	Post-abortion care	–	–	–	–	2 of 7	2 of 7	2 of7
	Antenatal care visit (1+) (contact)	NA	NA	84 %	–	NA	NA	NA
	Antenatal care visit (4+) (contact)	NA	NA	57 %	–	NA	NA	NA
	Essential package^b^	NA	NA	–	–	4 of 7	4 of 7	3 of 7
	Iron and folic acid supplementation^c^	NA	NA	26 %	–	4 of 7	2 of 7	3 of 7
	Tetanus toxoid vaccination^d^	NA	NA	81 %	–	4 of 7	0 of 7	3 of 7
	Prevention of malaria with prophylactic antimalarials	NA	NA	27 %	–	4 of 7	4 of 7	4 of 7
	Prevention of malaria with ITN	NA	NA	37 %	–			
	Smoking cessation during pregnancy	–	–	–	–	0 of 7	0 of 7	0 of 7
	Detection and management of syphilis^e^	44 %	2 %	37 %	–	5 of 7	5 of 7	5 of 7
	Detection and management of HIV (including PMTCT)^f^	–	3 %	67 %	*17 %*	6 of 7	4 of 7	6 of 7
	Calcium supplementation for deficient women in pregnancy	–	–	–	–	1 of 7	0 of 7	0 of 7
	Aspirin for pre-eclampsia prevention			–	–	1 of 7	0 of 7	0 of 7
	Antihypertensive drugs for hypertension in pregnancy			–	–	3 of 7	0 of 7	1 of 7
	Anticonvulsants (i.e., MgSO_4_)	–	–	–	–	7 of 7	2 of 7	3 of 7
	External cephalic version	–	–	–	–	0 of 7	0 of 7	0 of 7
	Induction of labor after pPRoM	–	–	–	–	0 of 7	0 of 7	1 of 7
	Antibiotics for pPRoM	–	–	–	–	6 of 7	0 of 7	3 of 7
	Antenatal corticosteroids	–	–	–	–	4 of 7	0 of 7	1 of 7
Childbirth and postpartum care	Skilled attendant at birth (contact)	NA	NA	66 %	–	NA	NA	NA
	Facility delivery (contact)	NA	NA	63 %	–	NA	NA	NA
	Social support during childbirth	NA	NA	–	–	0 of 7	0 of 7	0 of 7
	Prophylactic antibiotics for cesarean section	–	–	–	–	5 of 7	1 of 7	3 of 7
	Cesarean section	–	–	10 %	–	3 of 7	2 of 7	4 of 7
	Uterotonics for prevention of hemorrhage	NA	NA	–	–	8 of 8	5 of 8	6 of 8
	Active management of the 3rd stage of labor	NA	NA	–	–	8 of 8	5 of 8	4 of 8
	Induction at ≥ 41 weeks of gestation	–	–	–	–	0 of 7	0 of 7	1 of 7
	Uterotonics for treatment of hemorrhage	–	–	–	–	8 of 8	2 of 8	5 of 8
	Manual removal of the placenta	–	–	–	–	4 of 7	2 of 7	3 of 7
	*Initiation or continuation of HIV therapy for HIV positive women* ^*f*^ *(see above)* ^α^	–	3 %	67 %	17 %	6 of 7	4 of 7	6 of 7
	*Family planning and advice for contraceptives* ^*a*^ *(See above)* ^α^	–	22 %	31 %	4.00 %	5 of 7	4 of 7	3 of 7
	*Screen for and initiate or continue antiretroviral therapy for HIV* ^*f*^ *(see above)* ^α^	–	3 %	67 %	17 %	6 of 7	4 of 7	6 of 7
	Treat maternal anemia^g^	–	40 %	–	–	0 of 7	0 of 7	0 of 7
	Detect and manage postpartum sepsis	–	–	–	–	0 of 7	1 of 7	1 of 7
Newborns and small and ill babies	Immediate thermal care	–	–	–	–	4 of 7	3 of 7	2 of 7
	Promotion and support for early Initiation of breastfeeding	NA	NA	50 %	–	1 of 7	4 of 7	3 of 7
	Promotion and provision of hygienic cord and skin care	NA	NA	–	–	1 of 7	2 of 7	2 of 7
	Neonatal resuscitation	–	–	–	–	6 of 7	3 of 7	6 of 7
	Newborn Immunization^h^	NA	NA	91 %/37 %	–	2 of 7	0 of 7	1 of 7
	Presumptive antibiotic therapy for newborns at risk of bacterial infection	–	–	–	–	2 of 7	1 of 7	2 of 7
	Case management of neonatal sepsis, meningitis, and pneumonia	–	–	–	–	2 of 7	1 of 7	2 of 7
	*Initiation of ART in babies born to HIV infected mother* ^*f*^ *(see above)* ^α^	–	3 %	67 %	17 %	6 of 7	4 of 7	6 of 7
	Kangaroo mother care for preterm and for <2000 g babies	–	–	–	–	1 of 7	1 of 7	1 of 7
	Extra support for feeding the small and preterm baby	–	–	–	–	0 of 7	0 of 7	1 of 7
	Prophylactic and therapeutic use of surfactant	–	–	–	–	0 of 7	0 of 7	1 of 7
	Continuous positive airway pressure (CPAP) for RDS	–	–	–	–	0 of 7	0 of 7	1 of 7
	Management of newborns with Jaundice	–	–	–	–	1 of 7	0 of 7	1 of 7

^a^Screening outcome indicator is unmet need for family planning; coverage indicator is contraceptive prevalence rate; management outcome is total fertility rate

^b^Should include measurement of blood pressure, anaemia, and identification of pre-eclampsia

^c^indicator is percent of women taking 90+ days of iron supplementation, available from household surveys

^d^Source: data.unicef.org; defined as percent of neonates protected at birth from tetanus infection

^e^Source: WHO Global observatory; 56 countries with Process indicator, 62 with outcome, 31 with coverage able to be estimated

^f^Screening outcome is modelled results of HIV prevalence, available from aidsinfoonline.org/devinfo/libraries/aspx/Home.aspx; coverage indicator is percent of population in need with HIV managed, available from data.unicef.org; Management indicator is Paediatric transmission rate, available from data.unicef.org

^g^Anaemia prevalence in any woman of reproductive age, available from household surveys

^h^Source: data.unicef.org; BCG and Hepatitis B birth dose vaccinations

^i^The number of survey instruments which cover this topic out of all the survey instruments which could have covered this topic. Thus 7 survey instruments were identified for all topics except for haemorrhage for which 8 survey instruments were identified

^α^Duplicate indicators

The facility-based instruments did collect data on process indicators for many of the *Essential Interventions,* but were more focused on readiness to deliver antenatal care and emergency obstetric care; outcome indicators were rarely included. The availability of supplies was universally available from the survey instruments for only four interventions, while the supplies available for seven interventions were not tracked in any of the instruments. Similar results were observed for training relative to the *Essential Interventions* and for whether the intervention was actually performed. In addition, eight of the WHO *Essential Interventions* were not able to be tracked at facilities by any of the identified facility survey instruments (Table 2, facility data).

### Compilation and expert assessment

**Fig. 1:**
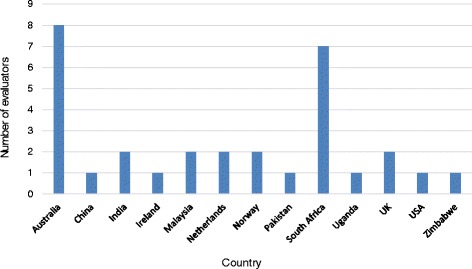
Countries represented by expert panel across scoring rounds (*N* = 31)

A total of 216 indicators were assembled across the following areas: 107 for preconception and antenatal care; 53 for childbirth and postpartum care; and 56 for care for newborns and small and ill babies. Of these, 122 were sourced or modified from existing indicators identified in instruments from available data sources and 94 were developed by the *eRegistries* technical team. Indicators were subsequently independently reviewed by evaluators. Response rates across the evaluation rounds were 23, 21, and 25 (68 %, 60 %, and 57 % respectively), with 31 evaluators in total participating (see Fig. 1 for distribution of countries represented). Some evaluators agreed to score a second set of indicators within the same round.

**Fig. 2:**
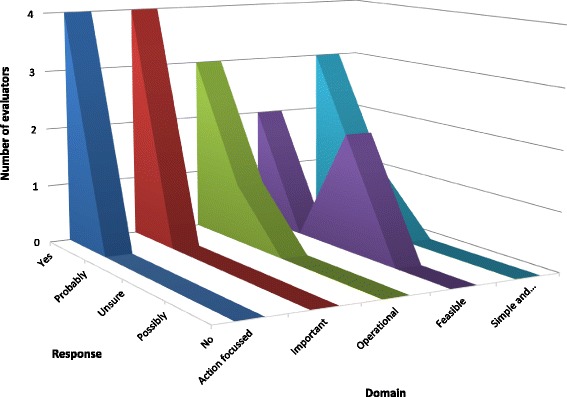
Evaluation for prophylactic antimalarial for preventing malaria in pregnancy treatment outcome indicator #3: *Malaria-specific stillbirth rate (per 100,000 births) (n = 4)*

Indicators for preconception/periconceptual care and antenatal care generally fulfilled the criteria ‘Action focused’, Important’, and ‘Simple and valued’. Indicators were less often deemed ‘Operational’ and ‘Feasible’, particularly for cause-specific mortality where capacity in some regions to accurately attribute death to specific conditions was considered lacking. The lack of availability of resources and skilled health personnel in LMIC settings was also seen as a barrier to the utility of many indicators. Figure 2 presents evaluation data for the treatment outcome indicator ‘Malaria-specific stillbirth rate (per 100,000 births)’, which represents the typical pattern of results received across evaluation rounds.

Indicators for childbirth care and postpartum care (of the mother) interventions were deemed to meet (or probably meet) all criteria by the majority of evaluators. Indicators consistently scored lowest on the criteria ‘Feasible’ and (less often) ‘Operational’, with variable evaluations on the criteria ‘Action focused’, ‘Important’, and ‘Simple and valued’. Panel members raised concerns around the feasibility of data collection in LMIC where many births occur at home and where follow-up is difficult. Difficulty accurately attributing cause of death for specific conditions/complications was again raised for all settings, but particularly in LMIC. The feasibility and appropriateness of the maternal near-miss indicators introduced in this round was questioned. A number of panel members thought that health workers may lack understanding and/or appreciation of the concept of near-miss, and some anticipated the indicators may lead to data manipulation in order to conceal what may be perceived as suboptimal care. Some of the indicators associated with the HIV interventions appeared too complex and needed clarification and simplification.

Indicators around newborn care received more mixed evaluations across the criteria, again, with feasibility a clear impediment. Evaluations varied on the criteria ‘Action focused’, ‘Important’, and ‘Simple and valued’.

#### Response to feedback and refinement within the eRegistries technical team

Amendments were made to a number of indicator titles, data points and definitions based on feedback from the expert panel (see Additional file 4 for examples). Amendments to indicators and definitions involved better operationalisation of key terms (e.g. breastfeeding counselling, ‘successful’ ECV, continuous support during labour). Indicators were reworded and redefined to improve direct measurability (e.g. Antenatal detection of breech presentation was revised to ‘Proportion of pregnant women who have presentation of baby checked by skilled birth attendant at or after 37 weeks of gestation’). Indicators and data points were also altered in some cases to ensure applicability in different settings; for example, the indicator measuring the proportion of women with postpartum haemorrhage who received therapeutic oxytocin was deemed too restrictive for settings where other uterotonics such as misoprostol are used.

Following refinements, there were a total of 193 unique indicators: 93 addressing preconception and antenatal care; 53 addressing childbirth and postpartum care; and 47 addressing care for newborns and small and ill babies Additional file 5 presents the final list of indicators including definitions, data points and sources (full indicator reports are available from the authors on request).

A series of meetings of the *eRegistries* technical team was held to review the updated indicators to ensure consistency in nomenclature across indicators and their data points, numerators and denominators. We established a set of definitions to guide the use of denominators and data points to ensure consistency across the indicator suite (Additional file 6).

### Display of utilisation of the indicators

**Fig. 3:**
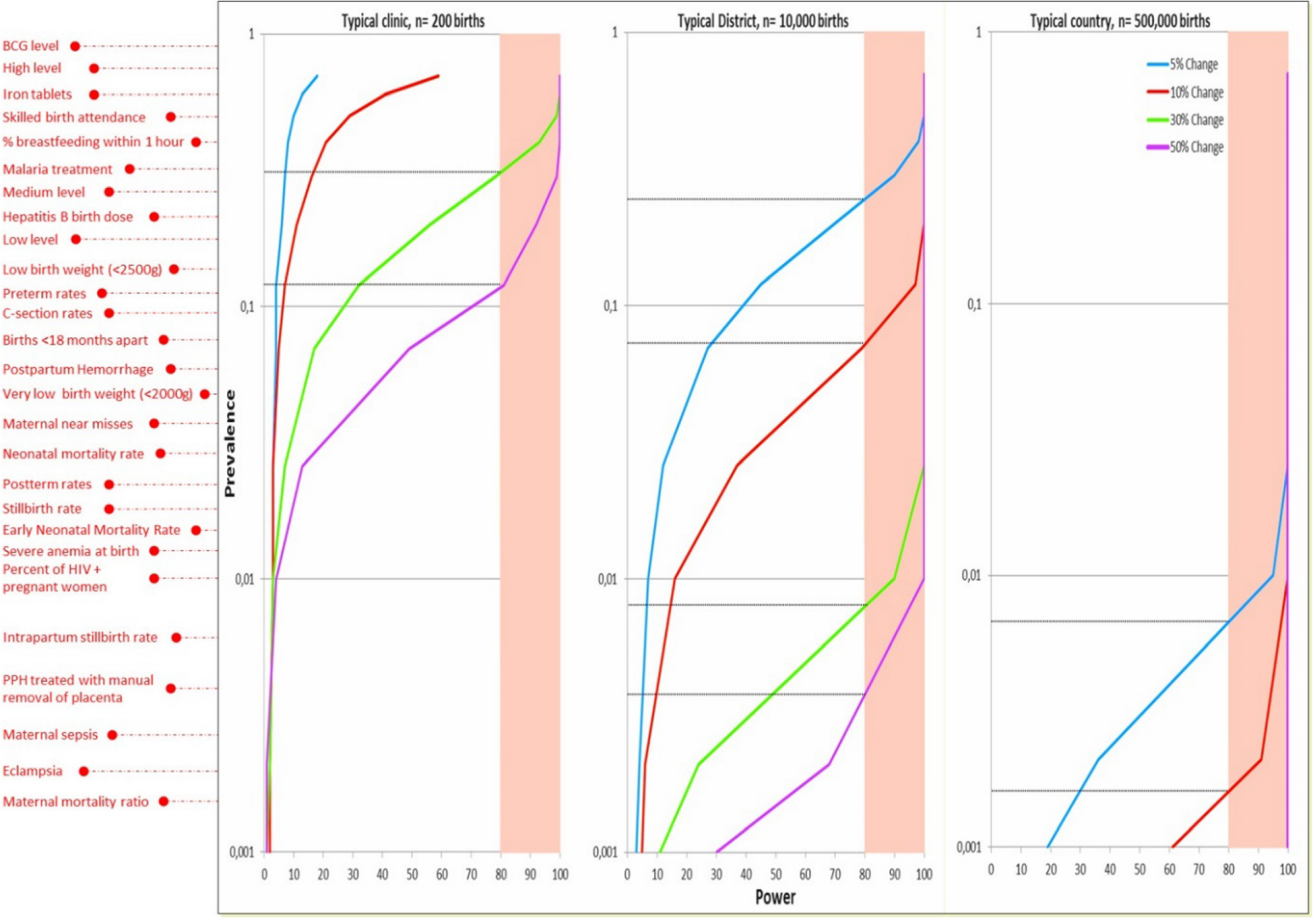
Correlation between indicator prevalence, statistical power and population size. Not all available indicators should be used equally when evaluating different levels of a health system. The selection of evaluation indicators should vary based on whether there is adequate power to measure a significant change and whether the outcome is modifiable by that level of the health system, a different set of indicators is appropriate for quality improvement at clinics than for national planning purposes. The figure indicates that a typical clinic (e.g. a facility with 200 births per year) only has adequate power to identify large differences in relatively frequent events, such as process indicators for interventions needed universally (i.e. SBA, immediate breastfeeding). Districts (10,000 births annually) have the ability to monitor relatively rare process indicators (management of maternal haemorrhage) as well as relatively common impacts (i.e. stillbirths) while populations the size of a typical country (e.g. 500,000 births annually) are needed to monitor rare outcomes (i.e. cause specific maternal mortality ratios or early neonatal mortality rates). All levels of the health system should be aware of the full range of indicators, but should only be evaluated on those which are appropriate at that specific level

Figure 3 demonstrates the association between statistical power, sample size and the prevalence of an indicator. As shown, there is likely to be insufficient power when measured at a clinic or district levels to determine differences in rare outcomes such as mortality, while adequate power would exist at national levels. In small clinics, there is likely to be sufficient power to measure differences in common management indicators, such as skilled birth attendance and iron supplementation.

## Discussion

We used the Donabedian framework [11] to determine the indicators required to successfully monitor the WHO *Essential Interventions*. This framework encompasses the intuitive relationship between three related concepts: first, the *structures* of health care are defined as the physical and organisational aspects of care settings (e.g., facilities, equipment, personnel); second, the *processes* of patient care in order to improve patient health; and, third, the well-known concepts of *outcomes* of medical care. Four groups of indicators were needed to clearly identify areas for quality improvement: process indicator/s for screening/risk identification (i.e. the proportion of screening tests/risk identification measures that were performed); outcome indicator/s for screening/risk identification (i.e. the proportion of women screening positive/identified as at risk); process indicator/s for treatment/management (i.e. the proportion of women treated); and outcome indicator/s for treatment/management (i.e. the proportion of adverse outcomes in the population). This work also drew upon health service ‘bottleneck analyses’ [13, 14], which consider structural and process factors that influence service delivery.

Our review of existing household and facility surveys which aredesigned to support national and global decision making, revealed a large gap in the availability of information to support the implementation of the WHO Essential Interventions, showing a critical need for improvements in data collection to monitor these interventions consistently across countries. Many of the needed indicators cannot be assessed retrospectively at the population level due to recall issues and lack of medical knowledge. On the other hand, many of the facility surveys focus narrowly on the structures of health care delivery. This leaves a large gap specifically in the arena of process indicators. In addition, the survey results and instruments evaluated demonstrate that several key interventions are not being monitored at all, even within the domains of structure or outcome. For example, there is no population level information to monitor antibiotics for pPRoM, while family planning activities and prevention and treatment of HIV are monitored relatively comprehensively. Although smoking cessation is recommended, none of the facility survey instruments included questions to assess this intervention and only one survey included questions on structural components to support the availability and utilization of calcium supplementation. These large gaps indicate that there is insufficient information available to guide countries and programs as they attempt to ensure the availability of the WHO *Essential Interventions* in their facilities and for their populations. There is a minimum of information to support the decision making at any of facility, national or global levels. It is this gap which the current indicator selection attempts to address.

We assembled a set of clearly defined process and outcome indicators, with a comprehensive suite of synergistic and consistent data points for effective measurement, for the 45 WHO *Essential Interventions* spanning preconception to newborn care, which can be collected from routine sources or facility surveys. Further, the *eRegistries* indicator evaluation tool makes a unique contribution to the metrics field, as it enables a priori assessment of the ‘likely’ or ‘potential’ utility of novel indicators. In our literature review, we found a paucity of information on indicator evaluation approaches to that did not rely on post-hoc review of the data generated by the indicator.

Feasibility in LMIC (informed by the direct experiences of many expert panel members) was a clear concern throughout the indicator evaluation process. Indeed, household surveys would, for example, not be able to assess all indicators proposed. Not all denominators would be measureable, making the results uninterpretable, and unattended home births would skew the numbers dramatically. However, the purpose of the proposed indicators is to facilitate uniform data collection in the presence of health workers documenting their actual work in a setting that accommodates an *eRegistry* or similarly-structured system for prospective data collection. Therefore, that a service (e.g. induction of labour for prolonged pregnancy) is not offered in a particular setting does not necessarily pose a problem for feasibility of data collection inherent in the indicator itself.

This study has several limitations. Although the expert panel consisted of members from low, middle and high-income settings, it did not include health systems specialists or programme managers, who may offer additional expertise. A legitimate feasibility problem may be posed by the use of cause-specific morbidity and mortality indicators, due to difficulty ascertaining cause of death, especially in some low-resource settings. This underscores the critical need for a quality, international, standardised cause of death classification system for maternal and perinatal mortality. Consistent application of the WHO’s International Classification of Diseases for Maternal Mortality and Perinatal Mortality (ICD-MM and ICD-PM) [23] may therefore enhance the utility of some of the indicators.

The indicators presented here are important for understanding the interplay between the patient and the health system, and how health system improvement can be achieved. The indicators presented do not represent an exhaustive list of the necessary indicators for all population-based or facility-based assessments, and should not be used in isolation. While these indicators are designed for use in settings with a functional health system collecting information on care-provision provision across the continuum of care, they are developed to minimise erroneous estimates in settings where a significant proportion of individuals do not attend a health system, and thus may not be included in denominators of the population. For example, where pregnancies in need of ANC are used in the denominator for ANC indicators, we define an eligible pregnancy as “a *woman having one documented ANC visit (unless the ANC visit is only for termination of pregnancy), OR any data documenting a pregnancy outcome or infant at any point of care”* (Additional file 6). This means that in an *eRegistry* setting tracking individuals, only individuals who are not in contact with any professional care provider from the health system through pregnancy, childbirth, post-partum care, newborn and infant care including vaccinations, will be missed as a population denominator.

In addition, these indicators are not a comprehensive set of indicators required to monitor a health system. These indicators are those that can be *collected through an eRegistry* and are necessary to address the 45 WHO *Essential Interventions*. Additional indicators for other components of a health system are clearly needed, as the WHO list does not include all interventions delivered by a health system (e.g. the management of diabetes in pregnancy). Other data sources, and potentially other indicators, should be utilized to understand the full continuum of availability, coverage and quality of community based activities, not only as stand-alone activities but also linked with facility-based activities.

The development of the *eRegistries* indicators is part of a global push to increase quality of care through an increased focus on measurement. The WHO recently published their vision for quality of care for maternal and newborn health [24], drawing partially upon the frameworks discussed in this paper, and have begun developing metrics to address quality of care. Two global action agendas, (‘Strategies toward Ending Preventable Maternal Mortality’ (EPMM) and the ‘Every Newborn Action plan’ (ENAP)) are also in the process of finalizing consensus metrics to increase the global ability to measure quality of care. These strategies contributed to the development of UN Secretary General’s Global Strategy for Women’s Children’s and Adolescents’ Health (2016–2030) which addresses relevant issues across the health in order to attain the related SDGs.

The *eRegistries* indicators contribute to the Indicator and Monitoring Framework [25] developed to support monitoring progress in implementation of the Global Strategy for Women’s Children’s and Adolescents’ Health (2016–2030) [26]. ‘Quality indicators’ are needed for monitoring healthcare, whether for internal quality improvement or for external accountability [27]. The *eRegistries* technical team developed this set of indicators to provide guidance on core indicators that should be part of routine information systems to ensure that a comparable, consistent and comprehensive set of indicators is available to countries as they attempt to link their new electronic data collection systems with the older household survey-based data sources. Importantly, any implementation of an *eRegistry* must adhere to national guidelines, and include careful development of customised indicators and associated data points. This set of indicators should serve as a warehouse or library of indicators for use whenever possible to ensure comparability with WHO standards for care. These indicators should not be used en masse to monitor all levels of a health system equally. Different indicators will have different uses as well as different characteristics, dependent on the size of the population which is being monitored and the type of decisions needed at various levels of the health system. As evidenced from the graphic display of the association between power, sample size and indicator prevalence, the selected indicators can vary dramatically in usefulness for the different levels of the health system. Indicators relevant for individual clinics will typically relate to those activities being undertaken at the clinic for all women. Small sample sizes related to rare complications and outcomes will make some indicators less useful for understanding practices within specific individual clinics, but collection of these data is nonetheless highly relevant for monitoring of public health at the national-level.

## Conclusions

With many countries in transition from paper to electronic data collection, more effort needs to be made to collect useable data at the point of data creation, and to minimise issues with recall, transcription and bias. The development of this suite of indicators can be used to support the implementation of *eRegistries* and other data platforms, to ensure that data are utilised to support evidence-based practice, facilitate measurement and accountability, and improve maternal and child health outcomes.

## Additional files

Additional file 1: Example score sheet with indicator criteria. (DOCX 329 kb) Additional file 2: Evaluation rounds, interventions addressed with number of indicators and indicator sources. (DOCX 44.4 kb) Additional file 3: Likely indicator values for power comparison displayed in Fig. 3. (DOCX 35.8 kb) Additional file 4: Response to feedback – examples of indicator amendments. (DOCX 41.5 kb) Additional file 5: List of final indicators. (DOCX 45 kb) Additional file 6: Glossary of denominator units and definitions. (DOCX 37.9 kb)

## Abbreviations

ANC: Antenatal care
DHS: Demographic and health surveys
ECV: External cephalic version
ICD PM: International classification of diseases perinatal mortality
ISA: International stillbirth alliance
LMIC: Low- and middle-income countries
MICS: Multiple indicator cluster survey
PROM: Prelabour rupture of membranes
RMNCH: Reproductive, maternal, newborn and child health
SDG: Sustainable development goals
ToP: Termination of pregnancy
UHC: Universal health care
UNAIDS: Joint United Nations, Programme on HIV/AIDS
UNICEF: The United Nations Children's Fund
WHO: World Health Organisation
WHS: World health statistics
