# Supplementary figures and images for: eRegistries: indicators for the WHO Essential Interventions for reproductive, maternal, newborn and child health
Source: BMC Pregnancy Childbirth. 2016 Sep 30;16:293. doi: 10.1186/s12884-016-1049-y (PMC5045645; doi:10.1186/s12884-016-1049-y)

## Appendices

### Appendix I. Example score sheet with indicator criteria


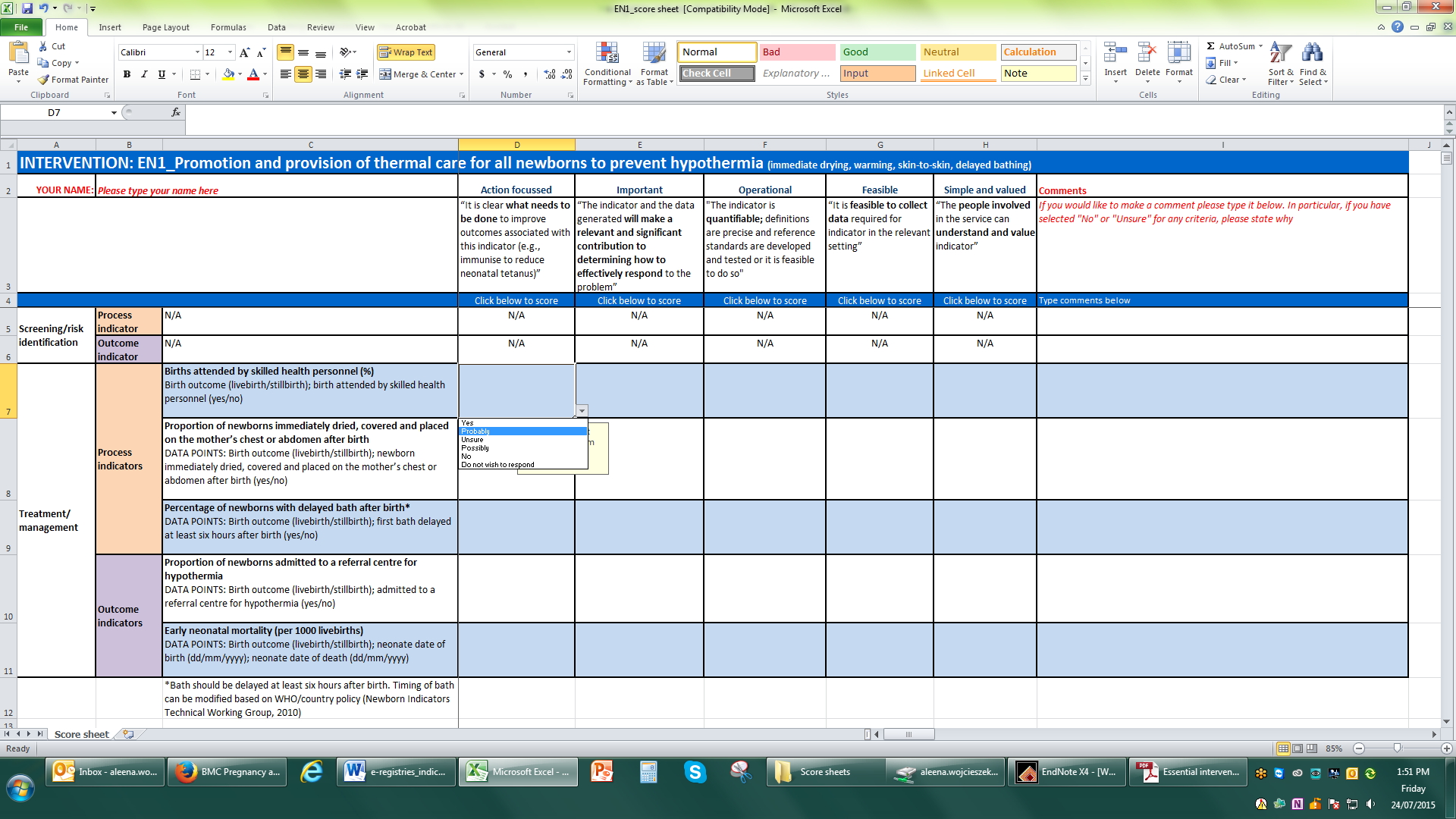

Supplement: Additional file 1: — Example score sheet with indicator criteria. (DOCX 329 kb) [file 12884_2016_1049_MOESM1_ESM.docx]
