# Supplementary material for: eRegistries: indicators for the WHO Essential Interventions for reproductive, maternal, newborn and child health
Source: BMC Pregnancy Childbirth. 2016 Sep 30;16:293. doi: 10.1186/s12884-016-1049-y (PMC5045645; doi:10.1186/s12884-016-1049-y)
Supplement: Additional file 2: — Evaluation rounds, interventions addressed with number of indicators and indicator sources. (DOCX 44.4 kb) [file 12884_2016_1049_MOESM2_ESM.docx]

### Appendix II. Evaluation rounds, interventions addressed with number of indicators and indicator sources

| **Interventions addressed** | | **Number of indicators** | **Indicator sources*** |
| --- | --- | --- | --- |
| **Round 1:**  **Preconception/periconceptual care and antenatal care (20 interventions)** | | 107 | WHO (51, 47%); eRegistries technical team (43, 40%); UNAIDS (4, 4%); RCOG (5, 5%); Other (4, 4%) |
| 1 | Family planning | 2 | NICE guidelines (1); WHO/UNICEF (1) |
| 2 | Prevention and management of STIs including HIV for PMTCT of HIV and syphilis | 7 | WHO (3); UNAIDS (1); Centers for Disease Control and Prevention (1); eRegistries technical team (2) |
| 3 | Folic acid fortification and/or supplementation to prevent neural tube defects | 2 | Centers for Disease Control and Prevention (1); eRegistries technical team (1) |
| 4 | Antenatal care essential package | 9 | WHO (6); eRegistries technical team (3) |
| 5 | Iron and folic acid supplementation during pregnancy | 6 | WHO (1); eRegistries technical team (5) |
| 6 | Tetanus immunisation in pregnancy for preventing neonatal tetanus | 4 | WHO (2); eRegistries technical team (2) |
| 7 | Prophylactic antimalarial for preventing malaria in pregnancy | 8 | WHO (6); eRegistries technical team (2) |
| 8 | Insecticide Treated Nets for preventing malaria in pregnancy | 6 | WHO (5); eRegistries technical team (1) |
| 9 | Smoking cessation during pregnancy | 4 | eRegistries technical team (4) |
| 10 | Screening and treatment of syphilis (during pregnancy) | 5 | WHO (4); Centers for Disease Control and Prevention (1) |
| 11 | Prevention and management of HIV and PMTCT in pregnancy | 4 | WHO (1); UNAIDS (3) |
| 12 | Calcium supplementation in pregnancy for preventing pre-eclampsia | 2 | eRegistries technical team (2) |
| 13 | Low-dose aspirin for preventing pre-eclampsia in high-risk women | 7 | WHO (3); eRegistries technical team (4) |
| 14 | Antihypertensive drugs for treating severe hypertension in pregnancy | 8 | WHO (4); eRegistries technical team (4) |
| 15 | Magnesium sulfate to prevent and treat eclampsia | 10 | WHO (6); eRegistries technical team (4) |
| 16 | External Cephalic Version (>36 weeks) to reduce malpresentation at term | 6 | WHO (3); RCOG (3) |
| 17 | Induction of labour for management of PROM at term | 6 | WHO (1); eRegistries technical team (5) |
| 18 | Antibiotics for management of pPROM | 5 | WHO (3); eRegistries technical team (1); RCOG (1) |
| 19 | Corticosteroids for prevention of respiratory distress syndrome in preterm labour | 4 | WHO (2); eRegistries technical team (1); RCOG (1) |
| 20 | Safe abortion for management of unintended pregnancy | 2 | WHO (1); eRegistries technical team (1) |
| **Round 2:**  **Childbirth care and postpartum care (of the mother) (12 interventions)** | | 53 | WHO (18, 34%); eRegistries working group (24, 45%); UNAIDS (5, 9%); Other (6, 12%) |
| 21 | Social support during childbirth | 3 | WHO (1); eRegistries technical team (2) |
| 22 | Prophylactic antibiotic for caesarean section | 3 | WHO (1); eRegistries technical team (2) |
| 23 | Caesarean section for maternal/fetal indication | 6 | WHO (5); eRegistries technical team (1) |
| 24 | Prophylactic uterotonic/active management of the third stage to prevent postpartum haemorrhage | 5 | WHO (2); eRegistries technical team (1); Health Services Research Group (2) |
| 25 | Induction of labour for prolonged pregnancy | 8 | WHO (4); eRegistries technical team (4) |
| 26 | Uterotonics for management of postpartum haemorrhage | 3 | WHO (2); eRegistries technical team (1) |
| 27 | Manual removal of the placenta (only by professional health workers) for management of postpartum haemorrhage | 3 | WHO (1); eRegistries technical team (2) |
| 28 | Initiation or continuation of HIV therapy for HIV positive women | 6 | UNAIDS (2); eRegistries technical team (4) |
| 29 | Advice and provision of family planning | 2 | Centers for Disease Control and Prevention (1); eRegistries technical team (1) |
| 30 | Prevent, measure and treat maternal anaemia | 4 | eRegistries technical team (4) |
| 31 | Detection and management of postpartum sepsis | 5 | RCOG (1); WHO (2); eRegistries technical team (2) |
| 32 | Screening and initiation or continuation of antiretroviral therapy for HIV | 5 | UNAIDS (3); eRegistries technical team (2) |
| **Round 3:**  **Immediate care of the newborn, Neonatal infection management, and care for small and ill babies (13 interventions)** | | 56 | WHO (23, 41%); eRegistries working group (27, 48%); Newborn Indicators Technical Working Group (TWG) (3, 5%), Other (3, 5%) |
| 33 | Promotion and provision of thermal care for all newborns to prevent hypothermia (immediate drying, warming, skin-to-skin, delayed bathing) | 5 | WHO (2); eRegistries working group (2); TWG (1) |
| 34 | Promotion and support for early initiation and exclusive breastfeeding (within the first hour) | 2 | WHO (2) |
| 35 | Promotion and provision of hygienic cord and skin care | 6 | WHO (3); eRegistries working group (1); TWG (2) |
| 36 | Neonatal resuscitation with a bag and mask for babies who do not breathe at birth | 2 | WHO (1); eRegistries working group (1) |
| 37 | Newborn immunisation | 4 | eRegistries working group (2); Tuberculosis Control Branch (1); Australian Government Department of Health and Ageing (1) |
| 38 | Presumptive antibiotic therapy for newborns at risk of bacterial infection | 4 | eRegistries working group (4) |
| 39 | Case management of neonatal sepsis, meningitis and pneumonia | 3 | WHO (2); eRegistries working group (1) |
| 40 | Initiation of ART in babies born to HIV-infected mothers | 2 | UNAIDS (1); eRegistries working group (1) |
| 41 | Kangaroo mother care (KMC) for preterm and for <2000g babies | 4 | WHO (3); eRegistries working group (1) |
| 42 | Extra support for feeding the small and preterm baby | 7 | WHO (2); eRegistries working group (5) |
| 43 | Prophylactic and therapeutic use of surfactant to prevent respiratory distress syndrome in preterm babies | 6 | WHO (4); eRegistries working group (2) |
| 44 | Continuous positive airway pressure (CPAP) to manage preterm babies with respiratory distress syndrome | 6 | WHO (4); eRegistries working group (2) |
| 45 | Management of newborns with jaundice | 5 | eRegistries working group (5) |

** Total percentages may not equal 100 due to rounding*
