# Supplementary material for: eRegistries: indicators for the WHO Essential Interventions for reproductive, maternal, newborn and child health
Source: BMC Pregnancy Childbirth. 2016 Sep 30;16:293. doi: 10.1186/s12884-016-1049-y (PMC5045645; doi:10.1186/s12884-016-1049-y)
Supplement: Additional file 3: — Likely indicator values for power comparison displayed in Fig. 3. (DOCX 35.8 kb) [file 12884_2016_1049_MOESM3_ESM.docx]

### Appendix III. Likely indicator values for power comparison displayed in Figure 3

| **Indicator** | **Value** | **Source** |
| --- | --- | --- |
| Maternal Mortality Ratio (MMR) | 0.00210 |  |
| Cause Specific Maternal mortality Ratio | 0.00057 | Applied Haemorrhage proportion from Say 2014 to global MMR |
| Neonatal Mortality Rate | 0.02000 | childmortality.org |
| Early Neonatal Mortality Rate | 0.01464 | childmortality.org * Oza |
| Stillbirth Rate | 0.01890 | Lawn 2015 |
| Intrapartum stillbirth rate | 0.00937 | Lawn 2015 |
| Preterm rates | 0.11800 | Lee |
| Low birthweight (<2500g) | 0.15500 | WHO 2004, Low birthweight: country, regional and global estimates |
| Very low birthweight (<2000g) | 0.06045 | 39% of low birthweight births are <2000g |
| Postterm rates | 0.02100 | Zeitlin 2007 |
| Postpartum haemorrhage | 0.06100 | Carroli |
| Eclampsia | 0.00350 | Souza 2010 |
| Maternal near misses | 0.03400 | Souza 2010 |
| PPH treated by manual removal of placenta | 0.00647 | Sheldon 2014 * PPH rates |
| Percent of HIV+ pregnant women | 0.01039 | SSA mean; unaidsinfoonline.org and UN pop births in 2005-2008 |
| Pregnant Anaemia rates | 0.41800 | <http://www.who.int/vmnis/database/anaemia/> anaemia_status_summary/en/ |
| Severe Anaemia at birth | 0.01400 | Vogel 2014 |
| Maternal sepsis | 0.00400 | Vogel 2014 |
| C-section rates | 0.08500 | DHS; global average |
| Iron tablets | 0.65700 | DHS; global average |
| malaria treatment | 0.55800 | DHS; global average |
| Births spaced less than 18 months apart | 0.07700 | DHS; adjusted to reflect the proportion of first births |
| Skilled birth attendance | 0.66000 | childinfo; DHS update |
| % breastfeeding within 1 hour | 0.43500 | DHS; global average |
| BCG vaccination rates | 0.91000 | data.unicef.org |
| Hepatitis B Birth dose for vaccination | 0.37100 | data.unicef.org |
| High level (sample coverage of routine activity) | 0.75000 |  |
| Medium level (sample coverage of routine activity) | 0.50000 |  |
| Low level (sample coverage of routine activity) | 0.25000 |  |
|  |  |  |
