# Supplementary material for: eRegistries: indicators for the WHO Essential Interventions for reproductive, maternal, newborn and child health
Source: BMC Pregnancy Childbirth. 2016 Sep 30;16:293. doi: 10.1186/s12884-016-1049-y (PMC5045645; doi:10.1186/s12884-016-1049-y)
Supplement: Additional file 4: — Response to feedback – examples of indicator amendments. (DOCX 41.5 kb) [file 12884_2016_1049_MOESM4_ESM.docx]

### Appendix IV. Response to feedback – examples of indicator amendments

| ***Intervention*** | ***Indicator*** | ***Comments*** | ***Summary of amendments*** |
| --- | --- | --- | --- |
| Prevention and management of STIs including HIV for PMTCT of HIV and syphilis | Proportion of women of reproductive age who are screened for sexually transmitted infections (screening process indicator) | *“Operational criteria: It is not clear which and how many STIs have to be screened for. Is this dependent on country specific guidelines? If so, it may be great to specify that”*  *“Will type of STI need to be specified for each case reported?”* | Indicator definition revised to: Proportion of women of reproductive age who are screened for sexually transmitted infections including HIV, and any other STIs relevant to regional or country-specific guidelines, of all sexually active women attending health services |
| Screening and treatment of syphilis (during pregnancy) | Proportion of pregnant women aged 15–24 years with a positive serology for syphilis attending antenatal care clinics (screening outcome indicator) | *“Why is this outcome indicator restricted to women between the ages of 15 and 24 years?”*  *“Why limit this to 15-24? Older women can get syphilis (younger than 15 as well)”*  *“Not clear to me why age-range has been selected - or that it would be meaningful in some communities”* | Indicator revised to: Proportion of pregnant women with a positive serology for syphilis |
| External Cephalic Version (>36 weeks) to reduce malpresentation at term | Antenatal detection of breech presentation (screening process indicator) | *“Checking occurs always, does not mean that the breech is also detected”* | Indicator revised to: Proportion of pregnant women who have presentation of baby checked by skilled birth attendant at or after 37 weeks of gestation |
| External Cephalic Version (>36 weeks) to reduce malpresentation at term | Proportion of women who have ‘successful’ ECV procedures (treatment outcome indicator) | *“It is not clear whether this outcome indicates cephalic presentation converted from ECV procedure is identified immediately after the procedure or at delivery. ‘Successful’ ECV procedure defined by cephalic presentation at delivery is more clinically important. I will change my agreement to 'Yes' if this outcome indicates a cephalic presentation at delivery, or 'Possibly' for a cephalic presentation before delivery.”* | Indicator revised to: Proportion of women who have ECV procedure performed by skilled birth attendant that result in conversion of breech to cephalic presentation at birth |
| Antibiotics for management of preterm prelabour rupture of membranes (pPROM) | Proportion of women with suspected preterm prelabour rupture of membranes (pPROM) for whom sterile speculum examination is performed (screening process indicator) | *“My concern here is that while a sterile speculum examination might be performed this does not mean that other interventions e.g., a VE have not been performed. Could rephrase to 'sterile speculum examination performed (only) to diagnose or outrule pPROM'”* | Indicator revised to: Proportion of women with clinical picture of pPROM for whom only sterile speculum examination is performed to diagnose or rule-out pPROM |
| Safe abortion for management of unintended pregnancy | Proportion of safe abortions of all abortions performed (treatment process indicator) | *“The use of this indicator is dependent on the number of abortions being known (this is the denominator) and by definition many of the unsafe abortions may not be carried out in registered clinics or by health professionals so this number may be very hard to quantify”*  *“in my view it is extremely difficult to collect reliable data on unsafe abortions for the denominator”* | Indicators removed as many unsafe abortions occur in unregistered clinics; the denominator is unattainable |
| Social support during childbirth | Proportion of labouring women who had continuous presence and support from a skilled or non-skilled person during labour and birth (treatment process indicator) | *“I think support may need to be explained… Most women need encouragement and some like physical support in terms of massage etc. So not easy to incorporate - maybe just to say '...continuous presence and active support...' would possibly be ok.”* | Indicator revised to: Proportion of labouring women who had continuous supportive presence during labour and birth. Indicator definition updated so that continuous presence was defined as presence throughout labour a birth, and not intermittent presence |
| Uterotonics for management of postpartum haemorrhage | Proportion of women with postpartum haemorrhage who received therapeutic oxytocin of all women with postpartum haemorrhage (treatment process indicator) | *“What about misoprostol?”*  *“Oxytocin, including Uniject is not available in all maternity units, especially in LMIC. In settings where oxytocin is not available, other uterotonics (i.e. ergometrine, prostaglandins including misoprostol) are strongly recommended to manage PPH (2011 WHO Recommendations for the prevention and treatment of PPH, strong recommendation, low-quality evidence). As the use of uterotonics other than oxytocin for management of PPH is not uncommon in reality, especially in LMIC, it would be very useful to have proportion of women with PPH who received any uterotonics (i.e. oxytocin and other uterotonics) reported as a process indicator”* | Indicator revised to: Proportion of women who received therapeutic uterotonics for management of postpartum haemorrhage |
| Promotion and support for early initiation and exclusive breastfeeding (within the first hour) | Proportion of pregnant women provided breastfeeding counselling in health facilities (treatment process indicator) | *“How is counselling to be defined? I know from other work I am involved in that this can be hugely challenging and can range from a telephone call to much more intensive programme of support.”*  *“My uncertainty here us around how breastfeeding counselling is defined, I know there are good ways but I think there are also poor ways. It is not clear how you will ensure this is good counselling. However, I think it is important to collect this information”*  *“The format of the antenatal information may be important. This might make a difference to whether the indicator impacts on the outcome. In addition the delivery of any one format might also make a difference”* | Indicator definition revised to include a definition of counselling as verbal and/or written information about the benefits and risks of breastfeeding provided by a trained maternity care professional during antenatal care |
