# Supplementary material for: eRegistries: indicators for the WHO Essential Interventions for reproductive, maternal, newborn and child health
Source: BMC Pregnancy Childbirth. 2016 Sep 30;16:293. doi: 10.1186/s12884-016-1049-y (PMC5045645; doi:10.1186/s12884-016-1049-y)
Supplement: Additional file 5: — List of final indicators. (DOCX 45 kb) [file 12884_2016_1049_MOESM5_ESM.docx]

Indicators – Full list

[Preconception interventions 4](#_Toc443316994)

[EPRE1_Family planning (preconception advice and counselling about choice of contraception) 4](#_Toc443316995)

[EPRE2_Prevention and management of STIs including HIV for PMTCT of HIV and syphilis 4](#_Toc443316996)

[EPRE3_Folic acid fortification and/or supplementation to prevent neural tube defects 4](#_Toc443316997)

[Antenatal care interventions 5](#_Toc443316998)

[EA1_Antenatal care essential package (four-visit schedule) 5](#_Toc443316999)

[EA2_Iron and folic acid supplementation during pregnancy 5](#_Toc443317000)

[EA3_Tetanus immunisation in pregnancy for preventing neonatal tetanus 5](#_Toc443317001)

[EA4a_Prophylactic antimalarial for preventing malaria in pregnancy 6](#_Toc443317002)

[EA4b_Insecticide Treated Bednets (ITBN) for preventing malaria in pregnancy 6](#_Toc443317003)

[EA5_Smoking cessation during pregnancy 7](#_Toc443317004)

[EA6_Screening and treatment of syphilis (during pregnancy) 7](#_Toc443317005)

[EA7_Prevention and management of HIV and prevention of mother-to-child-transmission (PMTCT) in pregnancy 7](#_Toc443317006)

[EA8a_Calcium supplementation in pregnancy for preventing pre-eclampsia 7](#_Toc443317007)

[EA8b_Low-dose aspirin for preventing pre-eclampsia in high-risk women 8](#_Toc443317008)

[EA8c_Antihypertensive drugs for treating severe hypertension in pregnancy 8](#_Toc443317009)

[EA8d_Magnesium sulfate to prevent and treat eclampsia 8](#_Toc443317010)

[EA9_External Cephalic Version (>36 weeks) to reduce malpresentation at term 9](#_Toc443317011)

[EA10a_Induction of labour for management of prelabour rupture of membranes (PROM) at term 9](#_Toc443317012)

[EA10b_Antibiotics for management of preterm prelabour rupture of membranes (pPROM) 10](#_Toc443317013)

[EA10c_Corticosteroids for prevention of neonatal respiratory distress syndrome (RDS) in preterm labour 10](#_Toc443317014)

[EA11_Safe abortion for management of unintended pregnancy 11](#_Toc443317015)

[Interventions for care at birth 11](#_Toc443317016)

[EC1_Social support during childbirth 11](#_Toc443317017)

[EC2_Prophylactic antibiotic for caesarean section 11](#_Toc443317018)

[EC3_Caesarean section for maternal/fetal indication (e.g., obstructed labour and central placenta previa) 11](#_Toc443317019)

[EC4_Prophylactic uterotonic/active management of the third stage to prevent postpartum haemorrhage 12](#_Toc443317020)

[EC5_Induction of labour for prolonged pregnancy 12](#_Toc443317021)

[EC6a_Uterotonics for management of postpartum haemorrhage 13](#_Toc443317022)

[EC6b_Manual removal of placenta (only by professional health workers) for management of postpartum haemorrhage 13](#_Toc443317023)

[EC7_Initiation or continuation of HIV therapy for HIV positive women 13](#_Toc443317024)

[Interventions for postpartum care (of the mother) 14](#_Toc443317025)

[EP1_Advice and provision of family planning 14](#_Toc443317026)

[EP2_ Prevent, measure and treat maternal anaemia 14](#_Toc443317027)

[EP3_Detection and management of postpartum sepsis 14](#_Toc443317028)

[EP4_ Screening and initiation or continuation of antiretroviral therapy for HIV 15](#_Toc443317029)

[Interventions for newborn care 15](#_Toc443317030)

[EN1_Promotion and provision of thermal care for all newborns to prevent hypothermia 15](#_Toc443317031)

[EN2_Promotion and support for early initiation and exclusive breastfeeding (within the first hour) 15](#_Toc443317032)

[EN3_Promotion and provision of hygienic cord and skin care 16](#_Toc443317033)

[EN4_Neonatal resuscitation with a bag and mask for babies who do not breathe at birth 16](#_Toc443317034)

[EN5_Newborn immunisation 16](#_Toc443317035)

[EN6_Presumptive antibiotic therapy for newborns at risk of bacterial infection 17](#_Toc443317036)

[EN7_Case management of neonatal sepsis, meningitis and pneumonia 17](#_Toc443317037)

[EN8_Initiation of ART in babies born to HIV-infected mothers 17](#_Toc443317038)

[Interventions for small and ill babies 18](#_Toc443317039)

[ESI1_Kangaroo mother care (KMC) for preterm and for <2000g babies 18](#_Toc443317040)

[ESI2_Extra support for feeding the small and preterm baby 18](#_Toc443317041)

[ESI3_Prophylactic and therapeutic use of surfactant to prevent respiratory distress syndrome in preterm babies 19](#_Toc443317042)

[ESI4_Continuous positive airway pressure (CPAP) to manage preterm babies with respiratory distress syndrome 19](#_Toc443317043)

[ESI5_Management of newborns with jaundice 19](#_Toc443317044)

| Preconception interventions | |
| --- | --- |
| EPRE1_Family planning (preconception advice and counselling about choice of contraception) | |
| *Screening process indicator* | N/A – no screening required |
| *Screening outcome indicator* | N/A – no screening required |
| *Treatment process indicator* | Proportion of women requiring contraception who have it documented in their notes that they have been informed about and offered a choice of all contraceptive methods, including LARC^[[1]](#endnote-1)^ methods |
| *Treatment outcome indicator* | Demand for family planning satisfied |
| EPRE2_Prevention and management of STIs^[[2]](#endnote-2)^ including HIV for PMTCT^[[3]](#endnote-3)^ of HIV and syphilis | |
| *Screening process indicator* | Proportion of women of reproductive age who were screened for sexually transmitted infections |
| *Screening outcome indicator* | Annual incidence of reported sexually transmitted infections among women of reproductive age (syndromic or etiological reporting) |
| *Treatment process indicator(s)* | Proportion of women engaging in safe sex |
|  | Proportion of women with sexually transmitted infections who are appropriately diagnosed, treated and counselled according to national guidelines |
| *Treatment outcome indicator(s)* | Proportion of infants born to HIV-infected mothers who test positive for HIV at 4-6 weeks |
|  | Proportion of newborns with congenital syphilis |
|  | Proportion of stillbirths attributable to syphilis |
| EPRE3_Folic acid fortification and/or supplementation to prevent neural tube defects | |
| *Screening process indicator* | N/A - intervention applies to all women of reproductive age |
| *Screening outcome indicator* | N/A - intervention applies to all women of reproductive age |
| *Treatment process indicator* | Folic acid intake: reproductive-aged women (percent) |
| *Treatment outcome indicator* | Proportion of births with neural tube defects |
| Antenatal care interventions | |
| EA1_Antenatal care essential package (four-visit schedule) | |
| *Screening process indicator* | N/A - intervention applies to all birthing women |
| *Screening outcome indicators* | N/A - intervention applies to all birthing women |
| *Treatment process indicator* | Proportion of pregnant women attending for four or more antenatal visits |
| *Treatment outcome indicator(s)* | Maternal mortality |
|  | Stillbirths |
|  | Low-birth-weight newborns |
|  | Preterm births |
|  | Early neonatal mortality |
| EA2_Iron and folic acid supplementation during pregnancy | |
| *Screening process indicator* | Proportion of women screened for severe anaemia of all pregnant women |
| *Screening outcome indicator* | Proportion of women identified antenatally with severe anaemia |
| *Treatment process indicator(s)* | Proportion of pregnant women given iron and folic acid supplements |
|  | Proportion of pregnant women with severe anaemia receiving therapeutic iron and folic acid supplements |
| *Treatment outcome indicator(s)* | Proportion of birthing women with severe anaemia |
|  | Proportion of birthing women with severe anaemia of all women diagnosed antenatally with severe anaemia |
| EA3_Tetanus immunisation in pregnancy for preventing neonatal tetanus | |
| *Screening process indicator* | Proportion of pregnant women whose vaccination status is checked at the first antenatal visit |
| *Screening outcome indicator* | Proportion of pregnant women who are sufficiently vaccinated against tetanus at the first antenatal visit |
| *Treatment process indicator* | N/A – covered by above screening outcome indicator |
| *Treatment outcome indicator(s)* | Neonates protected at birth against neonatal tetanus |
|  | Number of reported cases of neonatal tetanus |
| EA4a_Prophylactic antimalarial for preventing malaria in pregnancy | |
| *Screening process indicator* | N/A – applies to all women living in endemic areas with intense malaria transmission (stable malaria) |
| *Screening outcome indicator* | N/A – applies to all women living in endemic areas with intense malaria transmission (stable malaria) |
| *Treatment process indicator* | Birthing women living in endemic areas with intense malaria transmission (stable malaria) who have received malaria prophylaxis in the second and third trimester according to guideline |
| *Treatment outcome indicator(s)* | Proportion of maternal deaths among pregnant, birthing and postpartum women attributable to malaria |
|  | Proportion of newborns with congenital malaria |
|  | Proportion of stillbirths attributable to malaria |
|  | Proportion of early neonatal mortality attributable to malaria |
|  | Proportion of late neonatal mortality attributable to malaria |
| EA4b_Insecticide Treated Bednets (ITBN) for preventing malaria in pregnancy | |
| *Screening process indicator* | N/A – applies to all women living in endemic areas with intense malaria transmission (stable malaria) |
| *Screening outcome indicator* | N/A – applies to all women living in endemic areas with intense malaria transmission (stable malaria) |
| *Treatment process indicator* | Proportion of pregnant women living in endemic areas with intense malaria transmission (stable malaria) sleeping under insecticide treated bednets the night before the second antenatal visit |
| *Treatment outcome indicator(s)* | Proportion of maternal deaths among pregnant, birthing and postpartum women attributable to malaria |
|  | Proportion of newborns with congenital malaria |
|  | Proportion of stillbirths attributable to malaria |
|  | Proportion of early neonatal mortality attributable to malaria |
|  | Proportion of late neonatal mortality attributable to malaria |
| EA5_Smoking cessation during pregnancy | |
| *Screening process indicator* | Proportion of pregnant women whose smoking status was checked at the first antenatal visit |
| *Screening outcome indicator* | N/A – covered by above screening process indicator |
| *Treatment process indicator* | Proportion of pregnant women who received counselling on smoking cessation of those identified antenatally as currently smoking |
| *Treatment outcome indicator* | Proportion of women who were smoking at birth of those who reported currently smoking at the first antenatal visit |
| EA6_Screening and treatment of syphilis (during pregnancy) | |
| *Screening process indicator* | Proportion of pregnant women who were screened for syphilis at the first antenatal visit |
| *Screening outcome indicator* | Proportion of pregnant women with a positive serology for syphilis |
| *Treatment process indicator* | Proportion of syphilis-positive pregnant women receiving first-line antibiotic treatment by 24 weeks of gestation |
| *Treatment outcome indicator(s)* | Proportion of stillbirths attributable to syphilis |
|  | Proportion of newborns with congenital syphilis |
| EA7_Prevention and management of HIV and prevention of mother-to-child-transmission (PMTCT) in pregnancy | |
| *Screening process indicator* | Proportion of pregnant women who received testing and counselling at the first antenatal visit and received their results |
| *Screening outcome indicators* | N/A – covered by above screening process indicator |
| *Treatment process indicator* | Antiretroviral therapy coverage among HIV-infected pregnant women for PMTCT |
| *Treatment outcome indicator* | Proportion of infants born to HIV-infected mothers who test positive for HIV at 4-6 weeks |
| EA8a_Calcium supplementation in pregnancy for preventing pre-eclampsia | |
| *Screening process indicator* | N/A – preventative intervention applicable to all birthing women in areas where dietary calcium intake is low |
| *Screening outcome indicator* | N/A – preventative intervention applicable to all birthing women in areas where dietary calcium intake is low |
| *Treatment process indicator* | Proportion of pregnant women receiving calcium supplementation for preventing pre-eclampsia in areas where dietary intake is low |
| *Treatment outcome indicator* | Proportion of pregnant women with pre-eclampsia |
| EA8b_Low-dose aspirin for preventing pre-eclampsia in high-risk women | |
| *Screening process indicator* | Proportion of pregnant women who were screened for pre-eclampsia risk factors |
| *Screening outcome indicators* | Proportion of pregnant women identified antenatally at high-risk of pre-eclampsia |
| *Treatment process indicator* | Proportion of pregnant women at high-risk of pre-eclampsia receiving low-dose acetylsalicylic acid as the first option method of prevention of pre-eclampsia |
| *Treatment outcome indicator(s)* | Proportion of pregnant women with pre-eclampsia |
|  | Proportion of stillbirths attributable to pre-eclampsia |
|  | Proportion of early neonatal mortality attributable to preeclampsia |
|  | Proportion of late neonatal mortality attributable to preeclampsia |
| EA8c_Antihypertensive drugs for treating severe hypertension in pregnancy | |
| *Screening process indicator* | Proportion of women who had blood pressure measured at each antenatal visit |
| *Screening outcome indicators* | Proportion of pregnant women identified antenatally with hypertension |
| *Treatment process indicator* | Proportion of pregnant women with hypertension receiving antihypertensive drugs |
| *Treatment outcome indicator(s)* | Proportion of pregnant women with persistent hypertension of all women receiving antihypertensive drugs |
|  | Proportion of maternal deaths among pregnant, birthing and postpartum women attributable to hypertension |
|  | Proportion of stillbirths attributable to hypertension |
|  | Proportion of early neonatal mortality attributable to hypertension |
|  | Proportion of late neonatal mortality attributable to hypertension |
| EA8d_Magnesium sulfate to prevent and treat eclampsia | |
| *Screening process indicator* | Proportion of women who had blood pressure measured each antenatal visit |
| *Screening outcome indicators* | Proportion of pregnant women identified antenatally with severe preeclampsia |
| *Treatment process indicator(s)* | Proportion of women with severe preeclampsia or eclampsia treated with magnesium sulfate injection |
|  | Proportion of pregnant women with eclampsia receiving magnesium sulphate as the first option method of anticonvulsive therapy |
| *Treatment outcome indicator(s)* | Proportion of pregnant women developing eclampsia of all women presenting with severe pre-eclampsia |
|  | Proportion of pregnant women with eclampsia experiencing recurrence of convulsions of all women with eclampsia |
|  | Proportion of maternal deaths among pregnant, birthing and postpartum women attributable to eclampsia |
|  | Proportion of stillbirths attributable to eclampsia |
|  | Proportion of early neonatal mortality attributable to eclampsia |
|  | Proportion of late neonatal mortality attributable to eclampsia |
| EA9_External Cephalic Version (>36 weeks) to reduce malpresentation at term | |
| *Screening process indicator* | Proportion of pregnant women who have presentation of baby checked by skilled birth attendant at or after 37 weeks of gestation |
| *Screening outcome indicators* | N/A – covered by screening process indicator |
| *Treatment process indicator* | Proportion of women with a breech presentation at or after 37 weeks of gestation who are offered ECV^[[4]](#endnote-4)^ |
| *Treatment outcome indicator(s)* | Proportion of women who have ECV procedure performed by skilled birth attendant that result in conversion of breech to cephalic presentation at birth |
|  | Proportion of newborns with Stage 2 or Stage 3 HIE^[[5]](#endnote-5)^ after breech birth |
|  | Proportion of stillbirths attributable to complications of breech birth |
|  | Proportion of early neonatal mortality attributable to complications of breech birth |
|  | Proportion of late neonatal mortality attributable to complications of breech birth |
| EA10a_Induction of labour for management of prelabour rupture of membranes (PROM) at term | |
| *Screening process indicator* | Proportion of women with clinical picture of PROM^[[6]](#endnote-6)^ at term for whom only sterile speculum examination is performed to diagnose or rule-out PROM |
| *Screening outcome indicators* | Proportion of women with confirmed PROM at term |
| *Treatment process indicator(s)* | Proportion of women with confirmed PROM at term who are offered a choice of induction of labour with vaginal prostaglandin or expectant management |
|  | Proportion of women with confirmed PROM at term who undergo induction of labour with vaginal prostaglandin |
| *Treatment outcome indicator(s)* | Proportion of caesarean sections among women undergoing induction of labour with vaginal prostaglandin for PROM at term |
|  | Proportion of women with PROM at term with severe systemic infection or sepsis |
|  | Proportion of neonates with intrapartum (early-onset) sepsis born to women diagnosed with PROM at term |
| EA10b_Antibiotics for management of preterm prelabour rupture of membranes (pPROM) | |
| *Screening process indicator* | Proportion of women with clinical picture of pPROM^[[7]](#endnote-7)^ for whom only sterile speculum examination is performed to diagnose or rule-out pPROM |
| *Screening outcome indicators* | Proportion of women with confirmed pPROM |
| *Treatment process indicator* | Proportion of women diagnosed with pPROM receiving erythromycin for 10 days |
| *Treatment outcome indicator(s)* | Proportion of women with pPROM with severe systemic infection or sepsis |
|  | Proportion of stillbirths attributable to complications of pPROM |
|  | Proportion of early neonatal mortality attributable to complications of pPROM |
|  | Proportion of late neonatal mortality attributable to complications of pPROM |
| EA10c_Corticosteroids for prevention of neonatal respiratory distress syndrome (RDS) in preterm labour | |
| *Screening process indicator* | N/A – intervention applies to all preterm newborns |
| *Screening outcome indicator* | N/A – intervention applies to all preterm newborns |
| *Treatment process indicator* | Proportion of newborns delivered before 34+0 weeks of gestation exposed to antenatal corticosteroids |
| *Treatment outcome indicator(s)* | Proportion of newborns delivered before 34+0 weeks of gestation with respiratory distress syndrome |
|  | Proportion of early neonatal mortality attributable to RDS^[[8]](#endnote-8)^ or complications of RDS among preterm newborns |
|  | Proportion of late neonatal mortality attributable to RDS or complications of RDS among preterm newborns |
| EA11_Safe abortion for management of unintended pregnancy | |
| *Screening process indicator* | N/A |
| *Screening outcome indicator* | N/A |
| *Treatment process indicator* | N/A - ‘safe’ abortions cannot be measured |
| *Treatment outcome indicator* | Proportion of maternal deaths attributable to unsafe abortion |
| Interventions for care at birth | |
| EC1_Social support during childbirth | |
| *Screening process indicator* | N/A – applies to all birthing women |
| *Screening outcome indicator* | N/A – applies to all birthing women |
| *Treatment process indicator* | Proportion of labouring women who had continuous supportive presence during labour and birth |
| *Treatment outcome indicator(s)* | Proportion of birthing women given pain medication during labour and birth |
|  | Births by caesarean section |
| EC2_Prophylactic antibiotic for caesarean section | |
| *Screening process indicator* | N/A - intervention applicable to all women having a caesarean section |
| *Screening outcome indicator* | N/A - intervention applicable to all women having a caesarean section |
| *Treatment process indicator* | Proportion of women undergoing caesarean section and receiving prophylactic antibiotics |
| *Treatment outcome indicator* | Proportion of postpartum women with severe systemic infection or sepsis in the postpartum period of all women having caesarean sections |
| EC3_Caesarean section for maternal/fetal indication (e.g., obstructed labour and central placenta previa) | |
| *Screening process indicator* | Partograph use during labour and birth |
| *Screening outcome indicator* | Proportion of women with prolonged labour |
| *Treatment process indicator(s)* | Births by caesarean section for prolonged and/or obstructed labour |
|  | Caesarean sections after the onset of labour |
|  | Caesarean sections after the onset of labour in the absence of prolonged or obstructed labour |
| *Treatment outcome indicator(s)* | Intrapartum stillbirths |
|  | Early neonatal mortality in normally formed newborns |
|  | Early neonatal mortality attributable to prolonged and/or obstructed labour |
|  | Proportion of maternal near-misses attributable to uterine dysfunction, of all birthing women |
|  | Major perineal trauma |
| EC4_Prophylactic uterotonic/active management of the third stage to prevent postpartum haemorrhage | |
| *Screening process indicator* | N/A - intervention applicable to all birthing women |
| *Screening outcome indicator* | N/A - intervention applicable to all birthing women |
| *Treatment process indicator* | Proportion of women receiving oxytocin immediately after birth of the baby |
| *Treatment outcome indicator(s)* | Incidence of blood transfusions following postpartum haemorrhage after vaginal birth |
|  | Incidence of blood transfusions following postpartum haemorrhage after caesarean section |
|  | Proportion of maternal deaths attributable to postpartum haemorrhage |
|  | Proportion of maternal near-misses following postpartum haemorrhage of all birthing women |
| EC5_Induction of labour for prolonged pregnancy | |
| *Screening process indicator* | N/A – screening not applicable |
| *Screening outcome indicator* | N/A – screening not applicable |
| *Treatment process indicator(s)* | Proportion of women with uncomplicated pregnancies who are offered induction of labour between 41+0 and 42+0 weeks |
|  | Proportion of women with uncomplicated pregnancies who undergo induction of labour between 41+0 and 42+0 weeks |
| *Treatment outcome indicator(s)* | Births by caesarean section among prolonged pregnancies (41+) |
|  | Proportion of newborns with Stage 2 or Stage 3 Hypoxic Ischemic Encephalopathy following prolonged pregnancy (41+) |
|  | Proportion of newborns with meconium aspiration syndrome following prolonged pregnancy (41+) |
|  | Stillbirths following prolonged pregnancies among babies without major congenital abnormalities |
|  | Early neonatal mortality attributable to prolonged pregnancy (41+) or its complications |
|  | Late neonatal mortality attributable to prolonged pregnancy or its complications |
| EC6a_Uterotonics for management of postpartum haemorrhage | |
| *Screening process indicator* | N/A – screening not applicable |
| *Screening outcome indicator* | N/A – screening not applicable |
| *Treatment process indicator* | Proportion of women who received therapeutic uterotonics for management of postpartum haemorrhage |
| *Treatment outcome indicator(s)* | Proportion of maternal deaths as a result of postpartum haemorrhage |
|  | Proportion of maternal near-misses as a result of postpartum haemorrhage |
| EC6b_Manual removal of placenta (only by professional health workers) for management of postpartum haemorrhage | |
| *Screening process indicator* | N/A – screening not applicable |
| *Screening outcome indicator* | N/A – screening not applicable |
| *Treatment process indicator* | Proportion of women with postpartum haemorrhage and having a retained placenta for whom manual removal of the placenta is performed by a skilled birth attendant |
| *Treatment outcome indicator(s)* | Proportion of maternal deaths as a result of retained placenta and postpartum haemorrhage |
|  | Proportion of maternal near-misses as a result of uterine dysfunction with postpartum haemorrhage and retained placenta of all birthing women |
| EC7_Initiation or continuation of HIV therapy for HIV positive women | |
| *Screening process indicator* | Proportion of women presenting in labour with unknown serological status who were tested for HIV and received their results |
| *Screening outcome indicator* | Proportion of women newly diagnosed as HIV-positive during labour and delivery |
| *Treatment process indicator(s)* | Proportion of HIV-positive birthing women who received antiretrovirals to reduce risk of mother-to-child-transmission |
|  | Proportion of infants born to HIV-positive women receiving a virological test for HIV within 4-6 weeks of birth |
| *Treatment outcome indicator* | Proportion of infants born to HIV-infected mothers who test positive for HIV at 4-6 weeks |
| Interventions for postpartum care (of the mother) | |
| EP1_Advice and provision of family planning | |
| *Screening process indicator* | N/A – intervention applies to all birthing women |
| *Screening outcome indicator* | N/A – intervention applies to all birthing women |
| *Treatment process indicator* | Proportion of women who received counselling about contraceptive methods after the end of a pregnancy |
| *Treatment outcome indicator* | Proportion of pregnancies conceived within 18 months of a previous birth |
| EP2_ Prevent, measure and treat maternal anaemia | |
| *Screening process indicator* | Proportion of birthing women screened for anaemia in the postpartum period |
| *Screening outcome indicator* | Proportion of women identified postpartum with anaemia of all birthing women |
| *Treatment process indicator(s)* | Proportion of women with anaemia in the postpartum period who received iron supplementation |
|  | Proportion of postpartum women who received a blood transfusion for severe anaemia |
| *Treatment outcome indicator(s)* | Proportion of maternal deaths attributable to severe anaemia |
|  | Proportion of maternal near-misses attributable to coagulation/haematological dysfunction |
| EP3_Detection and management of postpartum sepsis | |
| *Screening process indicator* | Proportion of women receiving at least one health assessment within 48 hours after the end of pregnancy |
| *Screening outcome indicator* | Proportion of women with severe systemic infection/sepsis in the postpartum period |
| *Treatment process indicator* | Proportion of postpartum women with severe systemic infection or sepsis who were given antibiotics |
| *Treatment outcome indicator(s)* | Proportion of maternal deaths among postpartum women attributable to severe systemic infection or sepsis |
|  | Proportion of maternal near-misses attributable to severe systemic infection or sepsis |
| EP4_ Screening and initiation or continuation of antiretroviral therapy for HIV | |
| *Screening process indicator(s)* | Proportion of postpartum women with unknown serological status who were tested for HIV and received their results |
|  | Repeat screening and detection of HIV among postpartum women |
| *Screening outcome indicator* | Proportion of women newly diagnosed as HIV-positive in the postpartum period |
| *Treatment process indicator* | Proportion of women diagnosed with advanced HIV infection in the postpartum period who received antiretroviral therapy for their own health |
| *Treatment outcome indicator* | N/A – covered by treatment process indicator |
| Interventions for newborn care | |
| EN1_Promotion and provision of thermal care for all newborns to prevent hypothermia | |
| *Screening process indicator* | N/A – intervention applies to all newborns |
| *Screening outcome indicator* | N/A – intervention applies to all newborns |
| *Treatment process indicator(s)* | Proportion of births attended by a skilled birth attendant |
|  | Proportion of live newborns immediately dried, covered and placed on the mother’s chest or abdomen after birth |
|  | Proportion of live newborns with delayed bath after birth |
| *Treatment outcome indicator(s)* | Proportion of newborns admitted to a referral centre with hypothermia |
|  | Early neonatal mortality |
| EN2_Promotion and support for early initiation and exclusive breastfeeding (within the first hour) | |
| *Screening process indicator* | N/A – intervention applies to all newborns |
| *Screening outcome indicator* | N/A – intervention applies to all newborns |
| *Treatment process indicator* | Proportion of pregnant women provided breastfeeding counselling in health facilities |
| *Treatment outcome indicator* | Early initiation of breastfeeding |
| EN3_Promotion and provision of hygienic cord and skin care | |
| *Screening process indicator* | N/A – intervention applies to all newborns |
| *Screening outcome indicator* | N/A – intervention applies to all newborns |
| *Treatment process indicator(s)* | Proportion of newborns with cord cut with clean instrument |
|  | Proportion of newborns with nothing (harmful) applied to cord |
| *Treatment outcome indicator(s)* | Proportion of newborns with late-onset neonatal sepsis |
|  | Proportion of early neonatal mortality attributable to late-onset neonatal sepsis |
|  | Proportion of late neonatal mortality attributable to late-onset neonatal sepsis |
| EN4_Neonatal resuscitation with a bag and mask for babies who do not breathe at birth | |
| *Screening process indicator* | N/A – screening not applicable |
| *Screening outcome indicator* | N/A – screening not applicable |
| *Treatment process indicator* | Proportion of apnoeic live newborns receiving Positive Pressure Ventilation from health personnel trained in neonatal resuscitation |
| *Treatment outcome indicator* | Proportion of early neonatal mortality attributable to hypoxia |
| EN5_Newborn immunisation | |
| *Screening process indicator* | N/A - intervention applies to all newborns |
| *Screening outcome indicator* | N/A - intervention applies to all newborns |
| *Treatment process indicator(s)* | Proportion of newborns receiving BCG^[[9]](#endnote-9)^ immunisation within 24hrs of birth |
|  | Proportion of newborns receiving Hepatitis B (dose 1) immunisation within 24hrs of birth |
| *Treatment outcome indicator(s)* | N/A – beyond the scope of a reproductive health registry |
| EN6_Presumptive antibiotic therapy for newborns at risk of bacterial infection | |
| *Screening process indicator* | Proportion of live newborns screened for neonatal infection risk |
| *Screening outcome indicator* | Proportion of live newborns at risk for neonatal infection |
| *Treatment process indicator* | Proportion of newborns at risk of neonatal infection receiving presumptive antibiotic therapy |
| *Treatment outcome indicator(s)* | Proportion of newborns who develop intrapartum (early-onset) sepsis |
|  | Proportion of early neonatal mortality attributable to late-onset neonatal sepsis |
|  | Proportion of late neonatal mortality attributable to late-onset neonatal sepsis |
| EN7_Case management of neonatal sepsis, meningitis and pneumonia | |
| *Screening process indicator* | Proportion of live newborns screened for neonatal infection risk |
| *Screening outcome indicator* | Proportion of live newborns at risk for neonatal infection |
| *Treatment process indicator* | Proportion of live newborns with suspected or confirmed neonatal infection receiving antibiotics |
| *Treatment outcome indicator(s)* | Proportion of early neonatal mortality attributable to infection |
|  | Proportion of late neonatal mortality attributable to infection |
| EN8_Initiation of ART in babies born to HIV-infected mothers | |
| *Screening process indicator* | N/A – covered by previous interventions |
| *Screening outcome indicator* | N/A – covered by previous interventions |
| *Treatment process indicator(s)* | Proportion of live newborns born to HIV-infected women provided with antiretroviral prophylaxis to reduce the risk of early mother-to-child transmission in the first 6 weeks |
|  | Proportion of live newborns born to HIV-infected women who received a HIV PCR^[[10]](#endnote-10)^ test by 6 weeks of age |
| *Treatment outcome indicator* | Proportion of infants born to HIV-infected women who are diagnosed as HIV-positive by 6 weeks of age |
| Interventions for small and ill babies | |
| ESI1_Kangaroo mother care (KMC) for preterm and for <2000g babies | |
| *Screening process indicator* | Proportion of newborns who have gestation and birthweight recorded at birth |
| *Screening outcome indicator* | Proportion of newborns of <2000g birthweight and/or <34 completed weeks gestation |
| *Treatment process indicator* | Proportion of newborns of <2000g and/or <34 completed weeks gestation receiving KMC^[[11]](#endnote-11)^ |
| *Treatment outcome indicator(s)* | Early neonatal mortality among newborns of <2000g and/or <34 completed weeks gestation |
|  | Late neonatal mortality among newborns of <2000g and/or <34 completed weeks gestation |
| ESI2_Extra support for feeding the small and preterm baby | |
| *Screening process indicator* | Proportion of newborns who have gestation and birthweight recorded at birth |
| *Screening outcome indicator* | Proportion of newborns of <2000g birthweight and/or <34 completed weeks gestation |
| *Treatment process indicator* | Proportion of newborns of <2000g birthweight and/or <34 completed weeks gestation receiving human milk prior to discharge from health service |
| *Treatment outcome indicator(s)* | Proportion of newborns of <2000g birthweight and/or <34 completed weeks gestation with suspected or confirmed necrotising enterocolitis |
|  | Proportion of newborns of 2000g birthweight and/or <34 completed weeks gestation referred to an intensive care unit due to feeding problems |
|  | Proportion of newborns of 2000g birthweight and/or <34 completed weeks gestation with delayed discharge from birthing facility or extended stay in an intensive care unit due to feeding problems |
|  | Proportion of early neonatal mortality among newborns of 2000g birthweight and/or <34 completed weeks gestation attributable to malnutrition |
|  | Proportion of late neonatal mortality among newborns of 2000g birthweight and/or <34 completed weeks gestation attributable to malnutrition |
| ESI3_Prophylactic and therapeutic use of surfactant to prevent respiratory distress syndrome in preterm babies | |
| *Screening process indicator* | Proportion of newborns who have gestation recorded at birth |
| *Screening outcome indicator* | Proportion of newborns of <37 completed weeks gestation |
| *Treatment process indicator(s)* | Proportion of ventilator-dependent newborns of <30 weeks gestation at birth receiving prophylactic surfactant to prevent respiratory distress syndrome |
|  | Proportion of non-ventilator-dependent babies or babies of >30 weeks gestation at birth with established respiratory distress syndrome receiving therapeutic surfactant |
| *Treatment outcome indicator(s)* | Proportion of early neonatal mortality attributable to RDS or complications of RDS |
|  | Proportion of late neonatal mortality attributable to RDS or complications of RDS |
| ESI4_Continuous positive airway pressure (CPAP) to manage preterm babies with respiratory distress syndrome | |
| *Screening process indicator* | Proportion of newborns who have gestation recorded at birth |
| *Screening outcome indicator* | Proportion of newborns of <37 completed weeks gestation |
| *Treatment process indicator* | Proportion of preterm newborns with RDS receiving CPAP^[[12]](#endnote-12)^ |
| *Treatment outcome indicator(s)* | Proportion of newborns with chronic lung disease |
|  | Proportion of early neonatal mortality attributable to RDS or complications of RDS |
|  | Proportion of late neonatal mortality attributable to RDS or complications of RDS |
| ESI5_Management of newborns with jaundice | |
| *Screening process indicator* | Proportion of newborns screened for visible jaundice within 24hrs after birth of all live newborns |
| *Screening outcome indicator* | Proportion of newborns diagnosed with jaundice within 24hrs after birth of all live newborns |
| *Treatment process indicator* | Proportion of newborns with visible jaundice within 24hrs after birth receiving phototherapy |
| *Treatment outcome indicator(s)* | Proportion of newborns requiring exchange transfusion for neonatal jaundice |
|  | Proportion of newborns admitted to an intensive care unit for management of neonatal jaundice |

1. Long-acting reversible contraception [↑](#endnote-ref-1)
2. Sexually transmitted infections [↑](#endnote-ref-2)
3. Prevention of mother to child transmission [↑](#endnote-ref-3)
4. External cephalic version [↑](#endnote-ref-4)
5. Hypoxic ischemic encephalopathy [↑](#endnote-ref-5)
6. Prelabour rupture of membranes [↑](#endnote-ref-6)
7. Preterm prelabour rupture of membranes [↑](#endnote-ref-7)
8. Respiratory distress syndrome [↑](#endnote-ref-8)
9. Bacille Calmette-Guérin [↑](#endnote-ref-9)
10. Polymerase chain reaction [↑](#endnote-ref-10)
11. Kangaroo mother care [↑](#endnote-ref-11)
12. Continuous positive airway pressure [↑](#endnote-ref-12)
