# Supplementary material for: eRegistries: indicators for the WHO Essential Interventions for reproductive, maternal, newborn and child health
Source: BMC Pregnancy Childbirth. 2016 Sep 30;16:293. doi: 10.1186/s12884-016-1049-y (PMC5045645; doi:10.1186/s12884-016-1049-y)
Supplement: Additional file 6: — Glossary of denominator units and definitions. (DOCX 37.9 kb) [file 12884_2016_1049_MOESM6_ESM.docx]

### Appendix VI. Glossary of denominator units and definitions

| **Denominator unit** | **Definition and data points** | **Used for** |
| --- | --- | --- |
| Eligible pregnancy | A woman having one documented ANC visit (unless the ANC visit is only for ToP), OR any data documenting a pregnancy outcome or infant at any point of care | Indicators focussed on the woman and measured as part of ANC, regardless of birth outcome, where women attending ANC only for ToP should not be excluded (e.g. provision of iron and folic acid supplementation; smoking cessation counselling; screening for syphilis; HIV testing and counselling in pregnancy) |
| Birthing woman | Birthweight 500g, OR if missing; Gestation 22 weeks, OR if missing; Body length 25cm. For international comparisons: birthweight of 1000g, 28 weeks, 35cm. If all defining data points of birth are missing (Bodyweight, Gestation, Body length), then evidence of a live infant at any point of care. | Indicators focussed on women who have given birth, to assess quality of pregnancy, labour, birth and post-birth care (e.g. Proportion of pregnant women attending for four or more antenatal visits; Proportion of birthing women with severe anaemia; Fetal presentation screening at 37 weeks; Proportion of women with confirmed PROM at term) |
| Newborn | An infant with Birthweight 500g, OR if missing; Gestation 22 weeks, OR if missing; Body length 25cm. For international comparisons: 1000a, 28 weeks, 35cm. If all defining data points of birth are missing (Bodyweight, Gestation, Body length), then evidence of a live infant at any point of care | Indicators focused on babies and addressing pregnancy, labour, birth, and post-birth care, where babies who die before birth should be included (e.g., proportion of newborns with congenital malaria; low-birthweight newborns; proportion of stillbirths) |
| Live Newborn | Infant with Birthweight 500, OR if missing; Gestation 22 weeks, OR if missing; Bodylength 25cm) AND any sign of life. For international comparisons: (Birthweight 1000g, OR if missing; Gestation 28 weeks, OR if missing; Body length 35cm) AND any sign of life. If all defining data points of birth are missing (Bodyweight, Gestation, Body length, signs of life), then evidence of a live infant at any point of care | Indicators focused on babies and addressing pregnancy, labour, birth, and post-birth care, where babies who die before birth should not be included (e.g., neonates protected at birth against neonatal tetanus; early neonatal deaths; late neonatal deaths). Indicators assessing maternal deaths used a denominator of live newborns for consistency with established WHO indicators |

*ANC: Antenatal care; ToP: Termination of pregnancy; PROM: Prelabour rupture of membrane*
